# Global status of research on cutaneous squamous cell carcinoma and its programmed cell death: Bibliometric and visual analysis from 2012 to middle 2022

**DOI:** 10.3389/fonc.2023.1099382

**Published:** 2023-04-11

**Authors:** Yalin Zhang, Zhigang Guo, Huimin Wang, Bo Li

**Affiliations:** Medical School, Huanghe Science & Technology College, Zhengzhou, Henan, China

**Keywords:** cutaneous squamous cell carcinoma, knowledge graph, bibliometric analysis, CiteSpace, VOSviewer

## Abstract

**Objective:**

This study was aimed to analyze the research status and development trend of cutaneous squamous cell carcinoma (CSCC), focusing on the field of programmed cell death of CSCC, and providing suggestions for the research of CSCC.

**Methods:**

The publications related to CSCC and CSCC programmed cell death were searched in the Web of Science Core Collection (WOSCC) database, and timespan was set from 2012 to middle 2022. Research trends, authors, major country collaborations, research institutions, representative journals, publishers, and keywords were analyzed with CiteSpace and VOSviewer.

**Results:**

After screening, a total of 3656 publications on CSCC and 156 publications on CSCC cell programmed death were obtained. The number of published articles increased gradually with the years. The United States ranked first in terms of the number of published papers. Research in this field had focused on dermatology. Most of the institutions in both regions were from European and American countries. Harvard University was the most prolific institution. Wiley was the most prolific publisher. The popular keywords for programmed cell death in CSCC were cutaneous squamous cell carcinoma, diagnosis, pd 1, head, nivolumab and risk. Keywords in CSCC field were divided into 7 clusters: cutaneous squamous cell carcinoma, sentinel lymph node biopsy, skin cancer, B-Raf Proto-Oncogene, Serine/Threonine Kinase (BRAF) inhibitor and human Papillomaviruses, and P63 expression. Squamous cell carcinoma, cancer, head and expression were the most popular keywords. The popular keywords for programmed cell death in CSCC were cutaneous squamous cell carcinoma, diagnosis, pd 1, head, nivolumab and risk.

**Conclusion:**

This study analyzed the research status of cutaneous squamous cell carcinoma and programmed cell death from 2012 to middle 2022. Understanding research status and hotspots can help scholars, countries and policymakers to better understand the background and research frontier of CSCC, and guide further research directions.

## 1 Introduction

Cutaneous squamous cell carcinoma (CSCC) is a non-melanoma skin cancer, second only to basal cell carcinoma, accounting for 20% of skin cancers (1). The incidence of CSCCS is about 5 per 100,000, and the lifetime risk is 14-20%, which continues to increase each year (2). Statistics show that the incidence of CSCC has increased by at least 50% and even 200% in the past 30 years (3). These numbers are likely to continue to grow due to the ageing of the population and increasing awareness of these diseases.

Most CSCCS are formed by progressive progression of non-invasive precancerous lesions (4). CSCC results from genetic and epigenetic changes in cell replication during keratinosis. Over time, aggressive tumors accumulate and eventually form (5). When intracellular defensive abilities are impaired, the accumulation of genetic changes and the development of malignant and precancerous lesions are accelerated (6, 7). For example, free radical damage caused by reactive oxygen species, deamination or de-purine is the cause of CSCC (8, 9).

In order to maintain normal physiological homeostasis, cells die in various ways so that the body can get rid of aged and damaged cells. The most well-studied programmed cell death types, like apoptosis, necrotizing apoptosis, high-temperature death, ferroptosis, pan-apoptosis, and autophagy, have been found to play a crucial role in regulating the immunosuppressive tumor microenvironment and even in determining the efficacy of cancer therapy. Surgery and a combination of chemotherapy and radiation have long been considered the gold standard option for advanced squamous cell carcinoma. The studies of the therapeutic methods to inhibit programmed cell death provide a new direction to improve the therapeutic effect.

So far, there is still no efficient prevention and treatment strategies for CSCC, and the research directions of programmed cell death of CSCC are gradually coming into scholar’s vision. In order to comprehensively understand the research status and latest progress of CSCC, this paper adopts bibliometric method to analyze the publications related to CSCC and programmed cell death in WOSCC from 2012 to middle 2022. Combined with the two visualization tools, the main national cooperation, research institutions, representative articles, journals and publishers, research hotspots and frontiers in the field of CSCC and programmed cell death from 2012 to middle 2022 were reviewed, in order to provide suggestions for the research of CSCC.

## 2 Methods

### 2.1 Data collection

The literatures related to CSCC were searched in Core database based on WoSCC (https://www.webofscience.com/wos/woscc/basic-search). The data from WoSCC were comprehensive and standardized, and were widely used in bibliometrics. The retrieval strategies were “Cutaneous squamous cell carcinoma (All Fields) “ and “Cutaneous squamous cell carcinoma (All Fields) “. The literature type was limited to article, the language was limited to English, the time span was set from 2012 to middle 2022, and the retrieval time was June 30, 2022.

The full records and references of all literatures and the name, publication year, country/region, institution, source publication, research direction, author, key words, address, citation of each literature were downloaded from the WoSCC in TXT file format. All the literatures were selected separately by the experts of the two teams and finally included.

### 2.2 Bibliometric analysis tools

At present, the development and application of big data risk control and intelligent medical treatment all involve knowledge graph (10). Knowledge mapping is an outstanding visualization method research in information, scientometrics, knowledge rules and graphic expression in recent years (11). Up to now, knowledge graph has become one of the frontiers of visualization research in various fields. Among them, VOSviewer (12, 13), a visualization software developed by Van Eck and Waltman of Leiden University in the Netherlands, and CiteSpace (14–16), a visualization software developed by Chen Chaomei, a professor of computer and Information science at Drexel University in the United States, using the Java language, have attracted much attention. In this study, CiteSpace (Version 6.1.R2), VOSviewer (Version 1.6.10) and the literature statistics tools of WoSCC database were used to sort out and analyze CSCC related literatures, and verify the results of each other to ensure the accuracy of analysis. Set different analysis parameters according to different research contents.

CiteSpace is a common tool for mapping scientific knowledge. The aim is to visualize the structure, rules and distribution of scientific knowledge, and realize the mapping of author collaboration, keyword co-occurrence and institutional cooperation. Co-occurrence clusters, key nodes and research hotspots can be dynamically identified. At present, it has been widely used in the research hotspots and cooperative network analysis of medicine and health, humanities and philosophy, economic management, engineering and technology and other disciplines.

VOSviewer can also create visualizations. The difference is that the color of the circles represents different clusters, and the length of the line between the circles represents how close they are. Export the data of visual analysis, arrange the keywords according to the order of the average occurrence time, and get the current keywords; In the coauthored analysis, authors, institutions and countries are ranked according to link strength, and authors, institutions and countries with higher cooperative strength are obtained.

### 2.3 Main outcome indicators

The number of articles published by countries/regions, institutions, authors and journals, keyword co-occurrence and clustering, co-occurrence among authors, institutions and countries were the main analysis indicators.

## 3 Results

### 3.1 Annual trend of paper publication

After literature screening by 2 researchers, a total of 3656 literatures were finally included in this study. These literatures were cited for a total of 62359 times from 2012 to middle 2022, and the average citation times of each item was 17.06 and h-index was 89, that is, at least 89 literatures were cited for 89 times. Among them, 156 articles were about programmed cell death, and the average citation times of each item was 12.54 and h-index was 25.

**Figure 1:**
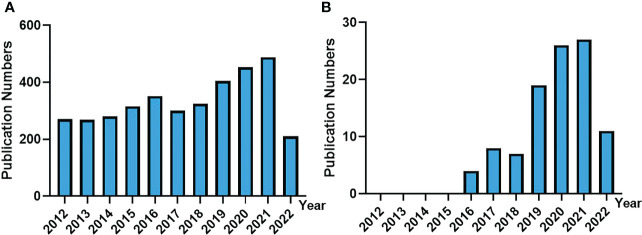
Number of literatures included in WoSCC from 2012 to 2022.**(A)**. Published literature on CSCC. **(B)**. Published literature on programmed cell death in skin cancer.

The number of published papers showed a wavy upward trend. In 2013, 268 articles were published, the lowest number in the last 11 years. Since then, the number of published papers has been increasing year by year, and it peaked at 480 in 2021, as shown in **Figure 1A**. In 2016, studies on programmed cell death of CSCC slowly came into view, and the number of studies involved in this field increased year by year and reached its peak in 2020, with 36 published in the whole year, and 100 articles related to programmed cell death of skin cancer were published from 2020 to middle 2022, accounting for 64.10% of the included literatures, as shown in **Figure 1B**.

The number and trend of published literatures play an important role in understanding the research heat and development trend of a field. According to the retrieval results, the annual number and percentage of CSCC publications in the world from 2012 to middle 2022 are shown in **Table 1**. In the past 11 years, the annual average number of articles published was more than 250, indicating that the research on CSCC has been the focus of scholars’ attention in the recent 11 years.

**Table 1:** Annual publication volume and percentage.

Rank	Publication Years	Record Count	% of 3,656
1	2021	480	13.129
2	2020	453	12.391
3	2019	405	11.078
4	2016	351	9.601
5	2018	324	8.862
6	2015	315	8.616
7	2017	300	8.206
8	2014	280	7.659
9	2012	270	7.385
10	2013	268	7.33
11	2022	210	5.744

### 3.2 Geographical distribution

**Figure 2:**
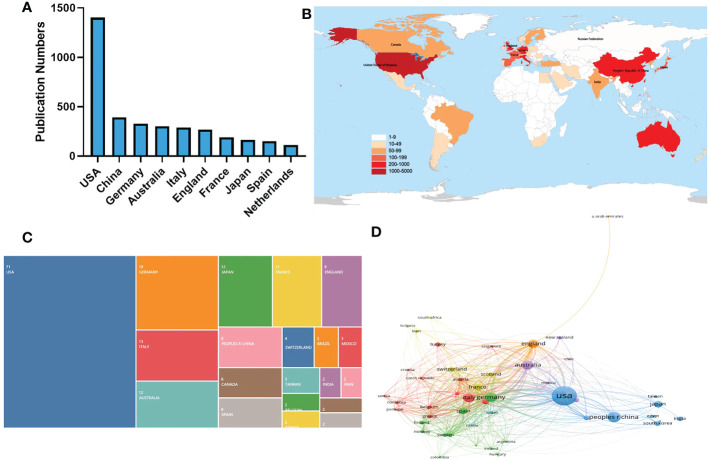
Published literature in countries/regions. **(A)**.The distribution trend of the top 10 countries/regions. **(B)**.Global map of countries/regions contributing to CSCC. **(C)**. National sequencing of CSCC cell programmed death studies. **(D)** Cooperation between countries in the CSCC field.

In terms of the number of publications, the United States ranked first with an amount of 1,403, accounting for 38.375% of the total number of punlications, which has an absolute advantage. The top five countries were China, Germany, Australia and Italy. More than 70 percent of the articles were published by the top five countries, as shown in **Figures 2A, B**. The country with the most publications on programmed cell death in skin cancer remains the United States, with 71 articles, followed by t Germany, Italy, Australia and Japan, as shown in **Figure 2C**.

Based on VOSViewer software, the geographic cooperation atlas of cutaneous squamous cell carcinoma research was established (**Figure 2C**), the nodes representing the United States are the largest, indicating that the United States is still the dominant country in this field. The next countries are China, the United Kingdom, Germany, Japan, etc., indicating that these countries have a certain research status in this field.

Using CiteSpace for statistical analysis, the first place is still the United States, with a centrality of 0.34, followed by the United Kingdom, Germany, France and Sweden, as shown in **Table 2**. In terms of centrality, the research results of the top ranked countries are more likely to be recognized by other countries.

**Table 2:** Number and centrality of published literatures in different countries.

Rank	Countries/Regions	Record Count	% of 3,656	Rank	Countries/Regions	Centrality
1	USA	1403	38.375	1	USA	0.34
2	PEOPLES R CHINA	394	10.777	2	ENGLAND	0.18
3	GERMANY	328	8.972	3	GERMANY	0.15
4	AUSTRALIA	303	8.288	4	FRANCE	0.1
5	ITALY	290	7.932	5	SWITZERLAND	0.09
6	ENGLAND	269	7.358	6	NETHERLANDS	0.08
7	FRANCE	191	5.224	7	SCOTLAND	0.08
8	JAPAN	166	4.54	8	PEOPLES R CHINA	0.06
9	SPAIN	151	4.13	9	ITALY	0.05
10	NETHERLANDS	113	3.091	10	SPAIN	0.05
11	SWITZERLAND	107	2.927	11	JAPAN	0.04
12	INDIA	100	2.735	12	NORWAY	0.04
13	BRAZIL	89	2.434	13	CZECH REPUBLIC	0.04
14	CANADA	86	2.352	14	AUSTRALIA	0.03
15	SOUTH KOREA	82	2.243	15	CANADA	0.03
16	SCOTLAND	76	2.079	16	SWEDEN	0.03
17	TURKEY	69	1.887	17	SAUDI ARABIA	0.03
18	AUSTRIA	55	1.504	18	AUSTRIA	0.02
19	FINLAND	53	1.45	19	DENMARK	0.02
20	SWEDEN	53	1.45	20	POLAND	0.02
21	DENMARK	50	1.368	21	MEXICO	0.02
22	NEW ZEALAND	49	1.34	22	BRAZIL	0.01
23	GREECE	48	1.313	23	GREECE	0.01
24	BELGIUM	42	1.149	24	BELGIUM	0.01
25	POLAND	40	1.094	25	PORTUGAL	0.01

### 3.3 Subject attribution analysis

**Figure 3:**
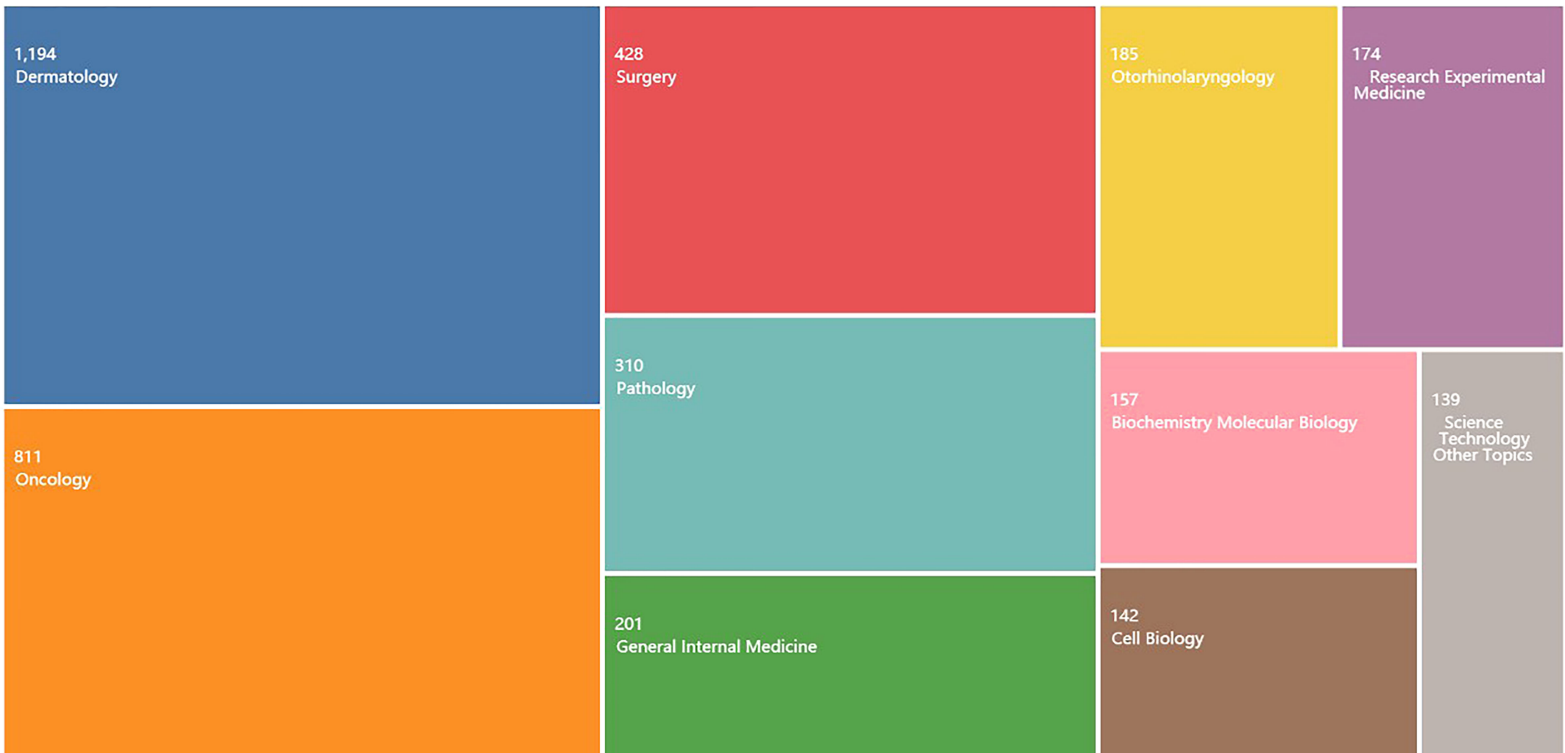
Analysis of research direction.

Studies in this field mainly focus on Dermatology (1194, 32.623%). The other main research directions were Oncology (811 articles, 22.158%), Surgery (428 articles, 11.694%), Pathology (310 articles, 8.470%), General Internal Medicine (201 articles, 5.492%). More than 80% of the literature on squamous cell carcinoma of the skin focuses were in the top 5 research directions, as shown in **Figure 3**.

### 3.4 Analysis of main institutions

**Figure 4:**
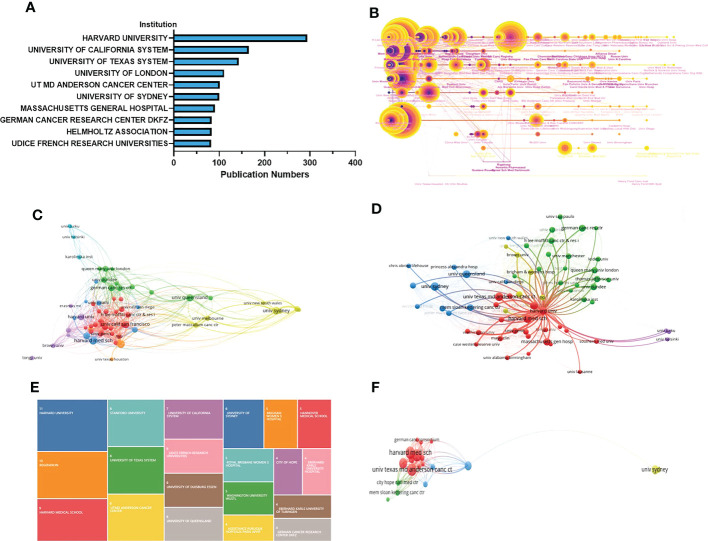
Literature analysis published by institutions. **(A)**. A statistical chart of the number of articles published by the top 10 institutions on CSCC research. **(B)**.Timeline and cluster view of all commonly referenced institutions. **(C)**.Excellent institutional coauthored analysis of the CSCC field. **(D)**.Excellent institutional co-citation analysis in the CSCC field. **(E)**.A statistical chart of the number of articles published by the top 20 institutions on CSCC cell programmed death studies. **(F)**.Excellent institutional coauthored analysis of the CSCC cell programmed death field.

Most of the top 25 institutions in the field of CSCC are from western countries. Among them, Harvard University published 294 papers, which is the institution with the most publications. The University of California System published 165 papers, and the University of Texas System published 143 papers, ranking second and third respectively. University of London, Ut Md Anderson Cancer Center and University of Sydney published more than 100 papers in this field, with 111, 101 and 100 papers respectively (**Figure 4A**; **Table 3**).

**Table 3:** Number and centrality of published literatures in different institution.

Rank	Institution	Record Count	% of 3,656	Rank	Institution	Centrality
1	Harvard University	294	8.042	1	Queen Mary University London	0.11
2	University of California System	165	4.508	2	University Modena & Reggio Emilia	0.09
3	University of Texas System	143	3.907	3	University Toronto	0.08
4	University of London	111	3.033	4	University Dundee	0.08
5	Ut Md Anderson Cancer Center	101	2.76	5	Mem Sloan Kettering Cancer Ctr	0.08
6	University of Sydney	100	2.732	6	German Cancer Res Ctr	0.08
7	Massachusetts General Hospital	91	2.486	7	University Sydney	0.07
8	German Cancer Research Center Dkfz	83	2.268	8	Massachusetts Gen Hosp	0.07
9	Helmholtz Association	83	2.268	9	University Calif San Francisco	0.06
10	Udice French Research Universities	82	2.24	10	Harvard University	0.06
11	University of California San Francisco	82	2.24	11	University Texas MD Anderson Cancer Ctr	0.05
12	Brigham Women S Hospital	81	2.213	12	University Calif Los Angeles	0.05
13	University of Queensland	78	2.131	13	Erasmus MC	0.05
14	State University System of Florida	70	1.913	14	Dana Farber Cancer Inst	0.05
15	Queen Mary University London	66	1.803	15	University Penn	0.04
16	H Lee Moffitt Cancer Center Research Institute	64	1.749	16	Kings Coll London	0.04
17	Memorial Sloan Kettering Cancer Center	61	1.667	17	Brigham & Womens Hospital	0.04
18	University of Pennsylvania	58	1.585	18	University Queensland	0.03
19	Institution National De La Sante Et De La Recherche Medical Inserm	56	1.53	19	University Miami	0.03
20	University of Dundee	56	1.53	20	University Arizona	0.03

According to the statistics of intermediary centrality, Queen Mary University London has the highest intermediary centrality (0.11), indicating that it is a hub connecting other institutions (**Figure 4B**).

Based on VOSviewer software, a collaboration atlas of CSCC research institutions was established. A total of 4,287 institutions were included in the study, and 63 institutions with more than 20 publications were selected to form the collaboration atlas, which was divided into 7 clusters. Among the institutions shown in **Figure 4C**, the total link strength value of the University of Sydney is 151, indicating that this institution has the most co-occurrence times with other institutions. As shown in **Figure 4D**, Massachusetts General Hospital has been cited the most times (6390 times), indicating that its literature publication quality is high and widely recognized.

In the field of programmed death of CSCC cells, Harvard University still Published the most papers (20), accounting for 12.82% of the total publications. *Nivolumab in Patients with Recurrent or Metastatic Squamous Cell Carcinoma of the Head and Neck: Efficacy and Safety in CheckMate 141 by Prior Cetuximab Use* is the most cited article on procedural death of skin cancer, with a total of 67 citations, published in *Clinical Cancer Research*, with an impact factor of 13.2. There are 19 institutions that have published more than 3 papers in this field. Except the University of Sydney, other institutions have close research exchanges. **Figures 4E, F**.

### 3.5 Principal author analysis

**Figure 5:**
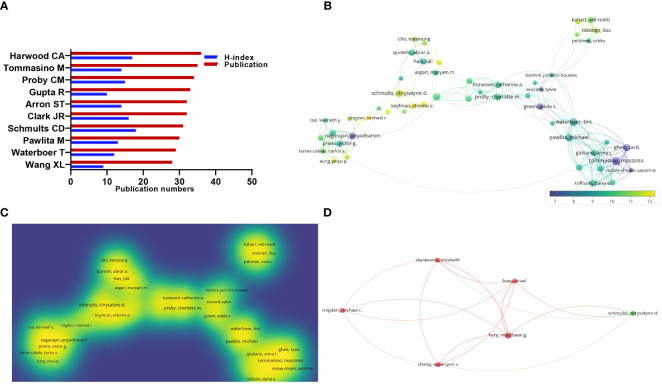
The author’s published literature. **(A)**. The number of CSCC articles published by the top 10 authors and the H-index. **(B)**. CSCC’s overlay visualization map of the top authors collaboration. **(C)**. CSCC’s density visualization map of the top authors collaboration. **(D)**. Excellent author’s co-citation analysis in the CSCC procedural death field.

Harwood CA (Queen Mary University London) has published 36 papers so far. Tommasino M (World Health Organization) ranks second with 35 publications. Proby CM (University of Dundee) ranked the third with 34 publications. The author with the highest H-index was Schmults CD (Harvard University), with a total of 31 publications, and the H-index was 18, that is, Schmults CD had at least 18 literatures being cited 18 times, as shown in **Figure 5A** and **Table 4**.

**Table 4:** The number of articles published by the author and h-index.

Author	publication	h-index
Harwood CA	36	17
Tommasino M	35	14
Proby CM	34	15
Gupta R	33	10
Arron ST	32	14
Clark JR	32	16
Schmults CD	31	18
Pawlita M	30	13
Waterboer T	29	12
Wang XL	28	9

Based on VOSviewer software, an author collaboration atlas of CSSS was established. A total of 18,426 authors had published relevant literatures, and 81 authors with more than 10 literatures were selected to form an institutional collaboration atlas, which was divided into 5 clusters. Among the authors shown in **Figure 5B**, the yellow cluster centered on Schmults CD (Harvard University), who was cited the most times. **Figure 5C** indicated that Schmults CD (Harvard University) has the highest density, suggesting that his literature is of high quality and is still recognized by other authors in this field. As shown in **Figure 5D**, there were only 6 scientists who published more than 5 articles. Fury and Matthew G were closely associated with other authors.

### 3.6 Analysis of journals and publishers

**Figure 6:**
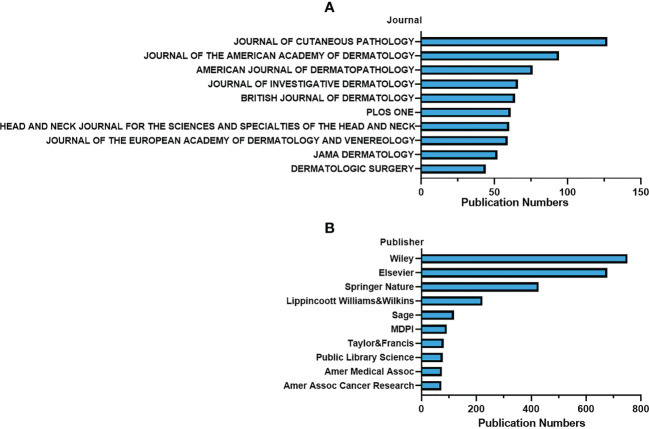
Statistic on the number of journals and magazines published. **(A)**.Statistical chart of the number of publications in journals. **(B)**.Statistical chart of the number of publications in publishers.

**Table 5** lists the top 25 journals and journals which had published the most relevant literatures in the field, as shown in **Figure 6**. The top 3 journals were *JOURNAL OF CUTANEOUS PATHOLOGY* (128 articles), *JOURNAL OF THE AMERICAN ACADEMY OF DERMATOLOGY* (95 articles) and *AMERICAN JOURNAL OF DERMATOLOGY DERMATOPATHOLOGY* (74 articles). The top three publishers were *Wiley* (751 articles), *Elsevier* (667 articles) and *Springer Nature* (426 articles), which published more than 50 percent of all publications.

**Table 5:** Journal and publisher statistics.

Rank	Publication Titles	Record Count	% of 3,661	Rank	Publishers	Record Count	% of 3,661
1	JOURNAL OF CUTANEOUS PATHOLOGY	128	3.496	1	Wiley	751	20.514
2	JOURNAL OF THE AMERICAN ACADEMY OF DERMATOLOGY	95	2.595	2	Elsevier	667	18.219
3	AMERICAN JOURNAL OF DERMATOPATHOLOGY	74	2.021	3	Springer Nature	426	11.636
4	JOURNAL OF INVESTIGATIVE DERMATOLOGY	67	1.83	4	Lippincott Williams & Wilkins	228	6.228
5	BRITISH JOURNAL OF DERMATOLOGY	63	1.721	5	Sage	116	3.169
6	PLOS ONE	61	1.666	6	Mdpi	87	2.376
7	HEAD AND NECK JOURNAL FOR THE SCIENCES AND SPECIALTIES OF THE HEAD AND NECK	60	1.639	7	Taylor & Francis	79	2.158
8	JOURNAL OF THE EUROPEAN ACADEMY OF DERMATOLOGY AND VENEREOLOGY	59	1.612	8	Public Library Science	69	1.885
9	JAMA DERMATOLOGY	53	1.448	9	Amer Medical Assoc	68	1.857
10	DERMATOLOGIC SURGERY	44	1.202	10	Amer Assoc Cancer Research	66	1.803
11	EXPERIMENTAL DERMATOLOGY	38	1.038	11	Frontiers Media Sa	62	1.694
12	INTERNATIONAL JOURNAL OF DERMATOLOGY	34	0.929	12	Karger	61	1.666
13	JOURNAL OF CUTANEOUS MEDICINE AND SURGERY	30	0.819	13	Spandidos Publ Ltd	59	1.612
14	SCIENTIFIC REPORTS	30	0.819	14	Oxford University Press	56	1.53
15	JOURNAL OF DERMATOLOGICAL SCIENCE	29	0.792	15	Bmj Publishing Group	34	0.929
16	JOURNAL OF DERMATOLOGY	28	0.765	16	Cureus Inc	32	0.874
17	CUREUS	26	0.71	17	Hindawi Publishing Group	32	0.874
18	ARCHIVES OF DERMATOLOGICAL RESEARCH	24	0.656	18	Wolters Kluwer Medknow Publications	32	0.874
19	INTERNATIONAL JOURNAL OF MOLECULAR SCIENCES	24	0.656	19	Int Inst Anticancer Research	28	0.765
20	ACTA DERMATO VENEREOLOGICA	23	0.628	20	E-Century Publishing Corp	26	0.71
21	ANTICANCER RESEARCH	23	0.628	21	Acta Dermato-Venereologica	23	0.628
22	FRONTIERS IN ONCOLOGY	23	0.628	22	Dove Medical Press Ltd	23	0.628
23	PHOTODIAGNOSIS AND PHOTODYNAMIC THERAPY	23	0.628	23	Thieme Medical Publishers	22	0.601
24	CLINICAL AND EXPERIMENTAL DERMATOLOGY	22	0.601	24	Edizioni Minerva Medica	21	0.574
25	JOURNAL OF DRUGS IN DERMATOLOGY	21	0.574	25	Journal Of Drugs In Dermatology	21	0.574

### 3.7 Research hotspot analysis

**Figure 7:**
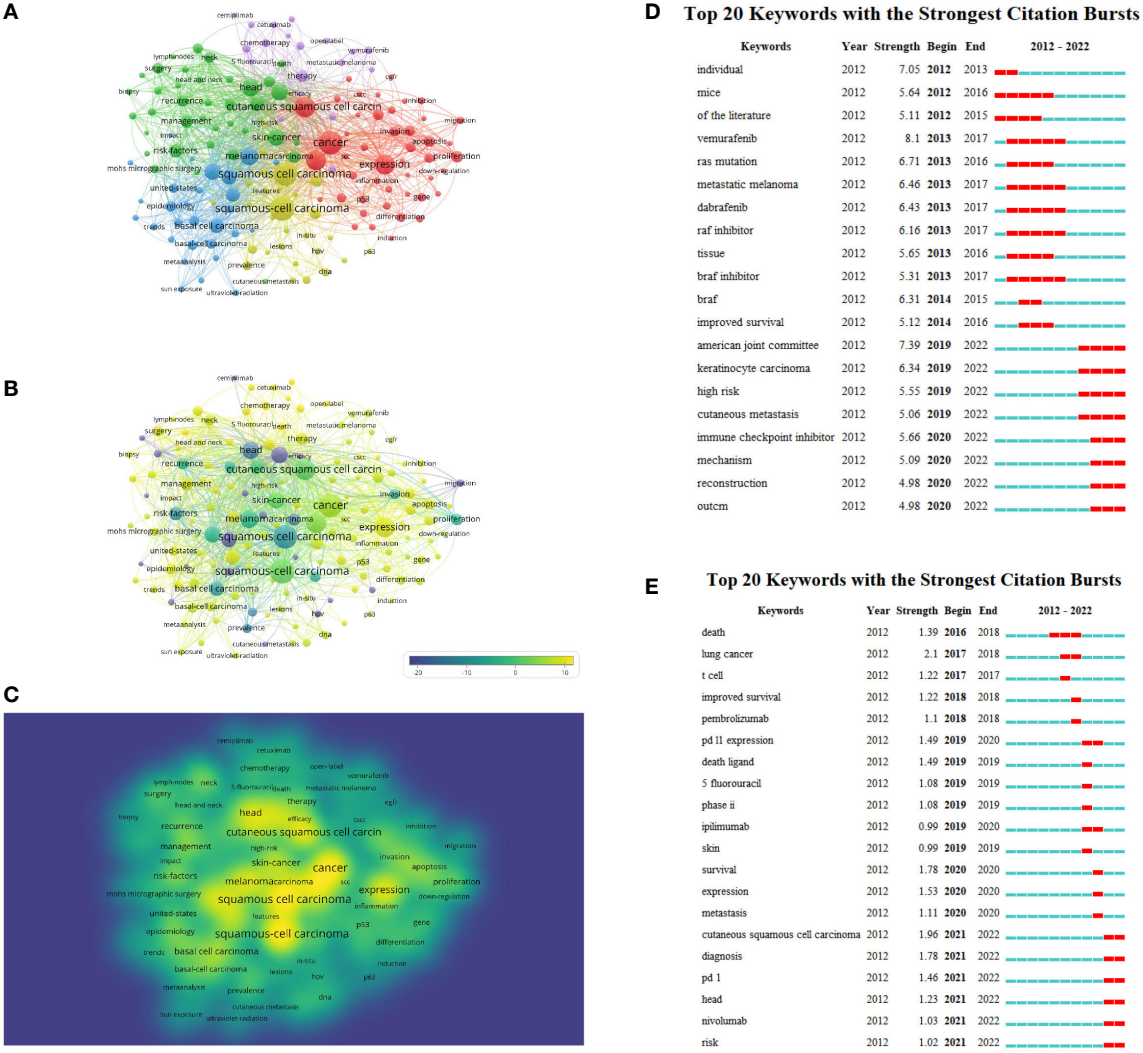
Keyword analysis diagram. **(A)**. Network visualization map of co-occurrence and clustering analysis of the frequent keywords. **(B)**.Co-occurrence and cluster analysis of frequent keywords in time-superimposed network visualization. **(C)**. Co-occurrence and cluster analysis of frequent keywords by density superposition network visualization. **(D)**.Keywords with the strongest citation bursts of CSCC. **(E)**. Keywords with the strongest citation bursts of programmed cell death in CSCC.

Keywords co-occurrence analysis was performed using VOSviewer software. 159 keywords out of 12,704 keywords were included in the network based on the inclusion criteria of more than 30 times of common occurrence, as shown in **Figure 7**. According to the keyword co-occurrence atlas, studies on squamous cell carcinoma of the skin were divided into 5 categories (**Figure 7A**): mechanism (red), treatment (purple), clinical (green), cancer (blue), and genetics (yellow). As shown in **Figure 7B**, the closer the circle is to green, the earlier the keyword appears. The closer the circle is to yellow, the later the keyword appears. **Figure 7C** is the density map of keywords. The closer the color of the keyword is to yellow, the higher the frequency of keyword occurrence. Squamous cell carcinoma, cancer, head and expression are highly popular keywords.

The word frequency growth algorithm based on CiteSpace can calculate the rapid growth of professional words in a short time and analyze the development trend of the research field of CSCC (**Figure 7D**). Before 2017, the keywords that popped up were individual, mice, of the literature, vemurafenib, ras mutation, metastatic melanoma, dabrafenib, Raf inhibitor, tissue, BRAF inhibitor and braf are mostly related to mechanism therapy and prognosis. Between 2019 and 2022, improved survival, American joint committee, keratinocyte carcinoma, high risk, cutaneous metastasis, immune checkpoint inhibitor, mechanism, reconstruction, outcm, most of which were related to the tumor. Through clinical randomized and retrospective studies, the prognostic factors affecting the survival rate of patients can be identified, which can help clinicians choose more reasonable treatment plans and improve the survival rate of patients.

The word spectrum of programmed cell death keywords in CSCC was completely different from that in CSCC. The hot keywords from 2021to middle 2022 were cutaneous squamous cell carcinoma, diagnosis, pd 1, head, nivolumab and risk. The keywords in 2022 were mostly related to immunotherapy. Clinical studies of both CSCC and programmed cell death have focused on therapy (**Figure 7E**).

**Figure 8:**
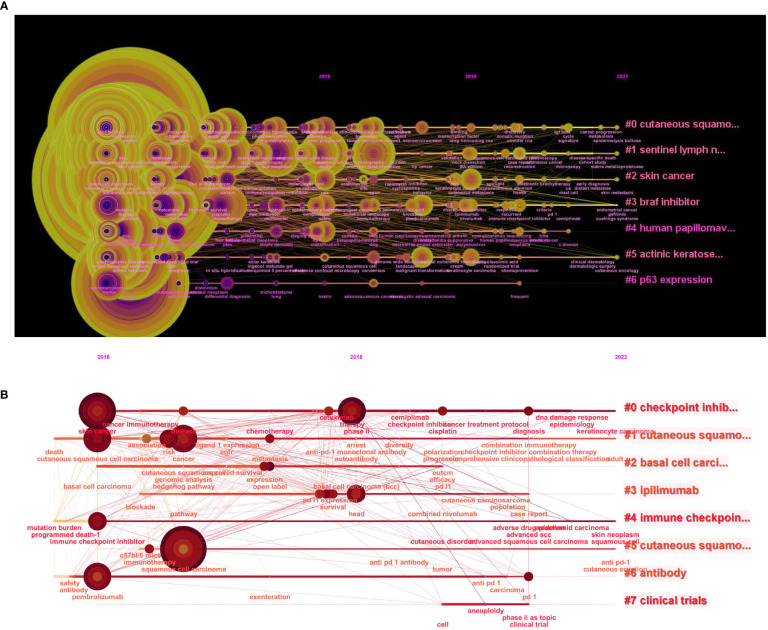
Keyword clustering atlas. **(A)**. Keyword clustering atlas of CSCC. **(B)**. Keyword clustering atlas in programmed cell death of CSCC.

According to the timespan cluster map generated by CiteSpace software (**Figure 8**), keywords in the field of CSCC from 2012 to middle 2022 can be divided into 7 clusters: cutaneous squamous cell carcinoma, sentinel lymph node biopsy, skin cancer, BRAF inhibitor, human papillomaviruses and human papillomaviruses and P63 expression. The size, average contour value and representative keywords of each cluster were shown in **Table 6**. The appendix shows the specific content of keyword clustering.

**Table 6:** Summary of the largest 7 clusters.

Cluster ID	Size	Silhouette	Label (LLR)	Average Year
0	171	0.661	cutaneous squamous cell carcinoma (11481.56, 1.0E-4)	2015
1	102	0.751	sentinel lymph node biopsy (7017.67, 1.0E-4)	2015
2	96	0.699	skin cancer (19537.04, 1.0E-4)	2015
3	84	0.719	braf inhibitor (4589.85, 1.0E-4)	2015
4	72	0.693	human papillomaviruses (4615.5, 1.0E-4)	2015
5	46	0.759	actinic keratoses (3305.52, 1.0E-4)	2015
6	24	0.881	p63 expression (1259.36, 1.0E-4)	2014

## 4 Discussion

CSCC is the second most common non-melanoma skin cancer, accounting for about 20% of skin cancers. However, a recent epidemiological study indicated that the ratio of basal cell carcinoma to squamous cell carcinoma in the medi-care population increased, even reaching 1:1 (17). According to data from the Mayo Clinic, the overall incidence of CSCC increased by 263% from 1976-1984 to 2000-2010 (3).

Bibliometrics is a statistical method, which can conduct quantitative analysis on research papers of a certain topic by using specialized software through mathematical statistics (18), analyzing key research areas and predicting future research directions.

CiteSpace is a Web-based Java application that can perform data analysis and visualization of massive data (19), which often provides a direction for the analysis and future development of the research field through the aspects of co-citation, co-author and common keywords (20). VOSviewer is a software for literature analysis and knowledge visualization developed by Leiden University Research Center for Science and Technology (21), which can realize network construction and visualization. Compared with other visualization software, VOSviewer has advantages for novices in processing big data and drawing images, and can more clearly display hot spots and topics in the research field without adjustment (22). CiteSpace and VOSviewer are important visualization tools for information visualization and metrological atlas research in recent years.

From 2012 to middle 2022, a total of 3,656 CSCC-related papers were published, and annual average of publications was more than 250. The number of publications showed a wavy upward trend. In the field of CSCC, the United States ranks first in the number of published documents, the degree of close contact between countries and the value of central mediation. The research in this field is mainly focused on Dermatology, and more than 80% of the literatures related to skin squamous cell carcinoma are concentrated in the top 5 research fields. Most of the institutions in the CSCC field are affiliated with western countries. Harvard University is the most published institution. The University of Sydney works closely with other institutions. Queen Mary University London has the highest level of central intermediation. Wiley was the magazine publishing the most magazines, and JOURNAL OF CUTANEOUS PATHOLOGY was the Journal publishing the most related articles.

Cutaneous squamous cell carcinoma research falls into five broad categories: mechanism, treatment, clinical, cancer and genetics. Squamous cell carcinoma, cancer, head and expression are highly popular keywords. Keywords in the CSCC domain can be divided into 7 clusters: Cutaneous squamous cell carcinoma, Sentinel lymph node biopsy, skin cancer, BRAF inhibitor, human papillomaviruses and human Papillomaviruses and P63 expression. These key clusters and star keywords suggest that, through the joint exploration of clinical and basic research, it is helpful for clinicians to select a more reasonable treatment plan and improve the survival rate of patients to clarify the prognostic factors affecting CSCC patients.

Immunotherapy is considered a breakthrough in the treatment of advanced CSCC. The available clinical evidence is supported by strong preclinical theory. Programmed cell death has become a hot topic in the field of CSCC. In the 10 years from 2012 to 2022, a total of 156 research literatures were published, and the literatures gradually increased in 2016. The two fields of CSCC and CSCC seem to contain a relationship, but there is a big gap between them. The field of CSCC’s programmed cell death is also centered on research institutions in western countries. Most studies are in the fields of immunotherapy and biotherapy, and the most prominent key word is PD-1. The most researches and treatments have been carried out on this protein. The study of programmed cell death in CSCC provides a bright development direction for CSCC therapy.

The content discussed in this topic is only a summary and induction of the literature published in the past, with limitations. Substantive research on CSCC programmed cell death will be carried out in clinical or scientific research in the future.

## 5 Conclusion

The study on the occurrence, development, treatment and prognosis of CSCC has become an important content in the tumor field. Programmed cell death studies in CSCC have led to key changes in advanced CSCC in terms of objective response, survival and quality of life improvement, and targeting the programmed cell death pathway could offer a concept of great promise for the treatment of CSCC.

## Data availability statement

The datasets presented in this study can be found in online repositories. The names of the repository/repositories and accession number(s) can be found in the article/supplementary material.

## Author contributions

ZG contributed to the conceptualization of the study. YZ played a part in the acquisition and analysis of data, and validation and visualization of results. HW participated in drafting and reviewing the main manuscript. BL contributed to the project administration, and funding acquisition. All authors contributed to the article and approved the submitted version.
